# An Intronic Variant in the *GRP78*, a Stress-Associated Gene, Improves Prediction for Liver Cirrhosis in Persistent HBV Carriers

**DOI:** 10.1371/journal.pone.0021997

**Published:** 2011-07-14

**Authors:** Xiao Zhu, Lianzhou Chen, Wenguo Fan, Marie C. M. Lin, Linwei Tian, Min Wang, Sheng Lin, Zifeng Wang, Jinfang Zhang, Jinlong Wang, Hong Yao, Hsiangfu Kung, Dongpei Li

**Affiliations:** 1 Cancer Institute, Affiliated Tumor Hospital, Guangzhou Medical University, Guangzhou, China; 2 Key Laboratory of Molecular Diagnosis, Institute of Biochemistry and Molecular Biology, Guangdong Medical College, Dongguan, China; 3 Department of Hepatobiliary Surgery, the First Affiliated Hospital, Sun Yat-Sen University, Guangzhou, China; 4 Zhongshan School of Medicine, Sun Yat-sen University, Guangzhou, China; 5 Department of Surgery, Prince of Wales Hospital, The Chinese University of Hong Kong, Hong Kong, China; 6 Department of Epidemiology, School of Public Health and Primary Care, The Chinese University of Hong Kong, Hong Kong, China; 7 Stanley Ho Center for Emerging Infectious Diseases, and Li Ka Shing Institute of Medical Sciences, The Chinese University of Hong Kong, Hong Kong, China; Ohio State University Medical Center, United States of America

## Abstract

**Background:**

Our previous study indicated that a common variant (rs430397 G>A) in the intron 5 of glucose-regulated protein 78 (*GRP78*) gene was associated with risk and prognosis of primary hepatocellular carcinoma (HCC), including HBV- and cirrhosis-related HCC. rs430397 polymorphism may be a contributing factor or biomarker of HBV infection or HBV-related cirrhosis.

**Methodology/Principal Findings:**

539 non-HBV-infected individuals, 205 self-limited infection and 496 persistent HBV infection were recruited between January 2001 and April 2005 from the hospitals in Southern China. Genomic DNA was genotyped for rs430397. The associations between the variation and susceptibility to liver cirrhosis (LC) in persistent HBV infection were examined. We observed that individuals carrying allele rs430397A were more likely to become HBV-related LC. When persistently infected patients were divided into four subgroups, patients with phase IV had an increased allele A and genotype AG compared with phase I and/or phase III. Decreased serum albumin and prolonged plasma prothrombin time (PT) were showed in LC patients carrying genotype AA. Furthermore, rs430397 genotype had an increased susceptibility to LC with dose-dependent manners (*P*-trend = 0.005), and the genotype did constitute a risk factor for the development of advanced LC (Child–Pugh classification C and B, *P*-trend = 0.021).

**Conclusions/Significance:**

rs430397 polymorphism may be a contributing factor to LC in persistent HBV carriers.

## Introduction

Hepatitis B, also called serum hepatitis, is a global health problem that affects more than 400 million of the world's population [1]. It is estimated that the prevalence of chronic hepatitis B virus (HBV) infection in China is approximately 10–20% of the population, where it represents the leading cause of cirrhosis and hepatocellular carcinoma [2]. Although HBV does not directly cause hepatic cytopathic effects, 15% to 40% HBV carriers will develop serious sequelae during their lifetime [1]. However, the mechanisms underlying the chronic HBV infection and its progression to different outcomes remain largely unsolved. Increasing evidences indicate that genetic factors might influence the natural history of chronic liver diseases [3], [4], [5], [6].

Like other viruses, HBV induce endoplasmic reticulum (ER) stress, which interrupts protein folding causing accumulation of unfolded or misfolded proteins in ER. To alleviate the stress placed on ER, these proteins must be refolded or degraded by activating a specific cellular response known as ER stress response or unfolded protein response (UPR) [7]. Glucose-regulated protein 78 (GRP78), which is a member of the HSP70 family of proteins and is found in the lumen of the ER is known to be a major cellular target of the UPR [8]. It not only binds to unfolded proteins but also regulates the activation of ER stress transducers such as IRE1, PERK, and ATF6 [9]. Based on these backgrounds, the transcriptional activation of the GRP78 is used extensively as a biological marker for onset of the UPR, as well as an unique model for deciphering the mechanisms whereby ER stress upregulates nuclear gene expression. With respect to liver disease, GRP78 may play an important role in HBV morphogenesis by regulating proper folding of the L protein and/or assembly of the envelope proteins [10]. *GRP78* has also been identified as a transformation-associated gene in hepatocellular carcinoma (HCC) [11].

HBV invasion and other physiopathologic changes cause large amount of unfolding or false-folding protein accumulation in the endoplasmic reticulum (ER), which in turn induces expression of GRP78 [12]. Our previous studies revealed that −87 T>A (a new mutation from the estimated translation start site of *GRP78* gene), the promoter and 3′ untranslated region (UTR) were not associated with HBV infection [13], [14]. Our recent studies showed that the 3′ UTR variants of *GRP78* were also not associated with risk and prognosis of HCC [15], [16]. But a common variant (rs430397 G>A) in the *GRP78* was found to be associated with risk and prognosis of primary HCC, including HBV- and cirrhosis-related HCC [17]. rs430397 is located in the intron 5 of *GRP78* and adjoin the 3′-end of the intron. Intronic mutation may be involved in DNA damage, mitogenic response and stress [18], [19]. Therefore, we hypothesized that rs430397 variant may also be a contributing factor to HBV infection and/or HBV-related cirrhosis. Accordingly, a large case-control study was conducted to further investigate this hypothesis among a *Han* Chinese population in Southern China.

## Materials and Methods

### Subjects and study design

The study protocol was approved by the Ethics Committee of Guangdong Medical College and adhered to the tenets of the Declaration of Helsinki. Additionally, written informed consent was obtained from each participant or each participant's guardian.

A total of 701 HBV-infected patients from southern China, a high-risk region of HBV infection, were recruited between January 2001 and April 2005. Of the 701 HBV-infected patients, 205 had self-limited infections with antibody to HBsAg-positive and antibody to HBcAg-positive, and 496 had persistent infections with HBsAg of the immunoglobulin G type in the serum for more than 36 months. All carriers were measured with liver function tests, serum immunologic marker screening, and liver ultrasonography. Some were screened by computed tomography (CT) and/or magnetic resonance imaging (MRI). Liver biopsies were performed in 103 patients, and the diagnosis of cirrhosis was made based on liver histology [20]. In patients without biopsy specimens, the diagnosis of cirrhosis was based on the presence of clinical manifestations of portal hypertension, ascites, biochemical evidences and obvious morphologic change of the liver detected by hepatic imaging. All carriers had no serologic evidence for coinfection with hepatitis C virus (HCV), hepatitis D virus (HDV), and human immunodeficiency virus (HIV). Patients with other types of liver diseases, such as autoimmune liver diseases, alcoholic liver diseases, or metabolic liver diseases, were also excluded. No evidence of tumors was found in patients with HBV after undergoing diagnostic procedures.

Patients with persistent infection, including 89 chronic HBV carriers (CHC) which based on sustained normalization of the serum alanine aminotransferase (ALT) levels together with seropositivity for HBeAg and high HBV-DNA levels throughout the study; 101 inactive hepatitis B surface antigen carrier (IHC) which has not shown significant, ongoing necroinflammatory disease; 108 chronic hepatitis B (CHB) which based on clinical symptoms, elevated serum aminotransferase levels, and/or abnormalities of other liver functions; and 198 liver cirrhosis (LC) which showed serum HBsAg, abnormal liver function profiles and portal hypertension, according to the guidelines of the American Association for the Study of Liver Diseases [21] and others [22], [23]. Child-Pugh score (CPS) was calculated in LC patients [24] and the LC patients were further divided into calss A (43), class B (87) and class C (68). According to the state of immune response, patients with persistent HBV infection can be classified as: phase I, immunotolerance phase, a chronic HBV carrier; phase II, immunoresponse phase, with HBeAg-positive chronic hepatitis or LC; phase III, an inactive HBsAg carrier and phase IV, with HBeAg-negative chronic hepatitis or LC [25], [26].

During the same time, 539 healthy adults, frequcncy-matched by gender and age (±6 years) group, from the above hospitals and other hospitals in Guangzhou region after physical checkup were enrolled as the control series. All the healthy controls were tested to exclude HBV, HCV, HIV infection and other conspicuous diseases. The main features of the subjects included are summarized in Table 1.

**Table 1:** Clinical and laboratory features of the subjects included in the study.

Characteristics	Non-HBV-infected Individualsn = 539	Self-limited Infectionn = 205	Persistent infection				
			Totaln = 496	CHCn = 89	IHCn = 101	CHBn = 108	LCn = 198
Age (years)							
Mean ± SD	43.0±9.7	38.1±9.0	41.9±13.5	34.8±9.5	37.9±10.3	42.5±10.6	43.9±11.8
Gender (%)							
Female	232 (43.04)	61 (29.76)	193 (38.91)	38 (42.70)	44 (43.56)	46 (42.59)	65 (32.83)
Male	307 (56.96)	144 (70.24)	303 (61.09)	51 (57.30)	57 (56.44)	62 (57.41)	133 (67.17)
Liver function test (mean±SD)							
T-Bil (µmol/L)	4.8±3.0	17.4±8.5	117.3±89.2	31.5±13.9	35.6±15.0	118.9±76.1	158.1±94.9
ALB (g/L)	44.6±5.9	34.6±7.0	31.2±6.7	32.1±6.1	33.4±4.9	30.7±5.8	25.7±6.5
ALT (IU/L)	26.7±11.4	27.1±9.8	161.1±95.5	30.7±10.4	32.4±15.9	263.8±180.4	160.9±118.1
AST (IU/L)	16.4±11.0	33.0±13.6	128.8±107.4	27.7±11.9	29.8±10.6	160.5±122.5	136.6±101.8
GGT (IU/L)	14.5±10.8	28.2±11.6	73.7±38.1	31.7±10.2	29.4±14.8	142.4±88.3	85.1±50.5
PT (seconds)	unknown	unknown	unknown	unknown	unknown	unknown	24.2±6.6
HBV serum tests (%)							
HBsAg (+)	0	0	496 (100)	89 (100)	101 (100)	108 (100)	198 (100)
HBeAg (+)	0	0	193 (38.91)	89 (100)	0	47 (43.52)	57 (28.79)
HBV-DNA (lg copies/mL)	0	0	4.88±2.31	4.96±1.15	3.55±0.91	4.62±2.29	5.94±1.80
Method of diagnosis (%)							
Histology			103 (20.77)	12 (13.48)	8 (7.92)	22 (20.37)	61 (30.81)
CT or MRI			80 (16.13)	3 (3.37)	5 (4.95)	14 (12.96)	58 (29.29)
Ultrasonic			496 (100)	89 (100)	101 (100)	108 (100)	198 (100)
Child-Pugh score (%)							
Class A							43 (21.72)
Class B							87 (43.94)
Class C							68 (34.34)

ALB, albumin; ALT, alanine aminotransferase; AST, aspartate aminotransferase; CHB, chronic hepatitis B; CHC, chronic hepatitis B virus carrier; CT, computed tomography; GGT, γ-glutamyltransferase; HBV, hepatitis B virus; IHC, inactive hepatitis B surface antigen carrier; LC, liver cirrhosis; MRI, Magnetic resonance imaging; PT, prothrombin time; SD, standard deviation;. T-Bil, total bilirubin.

### Resequencing and TaqMan genotyping

Resequencing and TaqMan genotyping were performed according to the methods described previously [17]. Briefly, Genomic DNA was extracted from peripheral blood leukocytes using QIAGEN QIAamp DNA Mini Blood Kit (Hilden, Germany) according to the manufacture's instructions. PCR was performed in a 50 µl reaction systems containing forward primer (AGTATTCTCGGTTGGCTGTTATG) and reverse primer (AGTTGCTGAATCTTTGGA). Resequencing was performed directly with the reverse primer, Taq polymerase, ABI PRISM® BigDye™ terminators on an ABI 3730×l DNA Analyzer (Applied Biosystems, Inc., Foster City, CA, USA). TaqMan high-throughput allelic discrimination assay was performed with a commercial kit (Applied Biosystems). The PCRs were run on the TaqMan Genotyping Master Mix without UNG (Applied Biosystems), with a PCR primer concentration of 500 nM and a TaqMan MGB-probe concentration of 200 nM. The reactions were carried out in a 96-well format in a total reaction volume of 20 µl using 50 ng of the genomic DNA. The plates were then placed in a thermal cycler (ABI 7500 Fast System, Applied Biosystems, Foster City, CA) and heated to 95°C for 10 min followed by 40 cycles of 92°C for 15 s and 60°C for 1 min.

### Statistical analysis

Mann–Whitney U-test was used to test the difference among the age groups. Chi-square test was used to determine whether there was a significant difference between groups. Hardy-Weinberg equilibrium (HWE) of genotype distribution among cases and controls was examined using the Pearson Chi-square test by Plink program [27]. Odds ratios (ORs) with 95% confidence intervals (95% CI) were computed by logistic regression to test the association of certain genotype with HBV infection and progression. We compared the minor allele genotypes to the most common genotype as a reference group. All statistical tests were 2-sided and *P* values less than 0.05 were considered statistically significant.

## Results

### rs430397 variant in individuals with non-HBV-infection, self-limited infection and persistent HBV infection

The genotype distributions of rs430397 G>A in these groups were in HWE. Meanwhile, a power analysis of the study population showed that our sample size was large enough to detect a possible association (date not shown). The genotyping results for individuals with non-HBV infection, self-limited infection and persistent HBV infection are shown in Table 2. Compared with individuals with non-HBV-infection or self-limited-infection, patients with LC were significantly more likely to carry allele A (*P* = 0.004 or *P* = 0.023). For the genotypes, the proportion of the AA genotype (6.57%) in patients with LC was different from that in non-HBV-infected individuals (3.15%; *P* = 0.018), while inversely, the frequency of GG genotype (60.10%) was significantly lower in LC patients compared to those without HBV infection (69.76%; *P* = 0.013). Although no statistical difference was found in allelic or genotypic frequencies among CHC, IHC, CHB and LC groups, a greater frequency of allele A (23.23%) or genotype AA (6.57%) in LC patients was found in comparison with the other groups. According to the Table 2, no statistical difference was found between the total persistent infection patients and the non-HBV-infected individuals or the self-limited infection patients (*P*>0.05, respectively).

**Table 2:** Genotype distribution of rs430397 G>A in the fifth intron of the *GRP78* gene in HBV-infected individuals with different clinical outcomes.

Variables	Non-HBV-infectedIndividualsn = 539, (%)	Self-limitedInfectionn = 205, (%)	Persistent infection				
			Totaln = 496, (%)	CHCn = 89, (%)	IHCn = 101, (%)	CHBn = 108, (%)	LCn = 198, (%)
Alleles							
G	898 (83.30)	341 (83.17)	812 (81.85)	155 (87.08)	173 (85.64)	180 (83.33)	304 (76.77)
A	180 (16.70)	69 (16.83)	180 (18.15)	23 (12.92)	29 (14.36)	36 (16.67)	92 (23.23)*
Genotypes							
GG	376 (69.76)	142 (69.27)	337 (67.94)	69 (77.53)	74 (73.27)	75 (69.44)	119 (60.10)†
AG	146 (27.09)	57 (27.80)	138 (27.82)	17 (19.10)	25 (24.75)	30 (27.78)	66 (33.33)
AA	17 (3.15)	6 (2.93)	21 (4.23)	3 (3.37)	2 (1.98)	3 (2.78)	13 (6.57)‡

CHB, chronic hepatitis B; CHC, chronic hepatitis B virus carrier; HBV, hepatitis B virus; IHC, inactive hepatitis B surface antigen carrier; LC, liver cirrhosis.

*Compared with the non-HBV-infected individuals, *x^2^* = 8.219 and *P* = 0.004; and compared with the self-limited infection, *x^2^* = 5.167 and *P* = 0.023.

† Compared with the non-HBV-infected individuals, *x^2^* = 6.124 and *P* = 0.013.

‡ Compared with the non-HBV-infected individuals, *x^2^* = 5.594 and *P* = 0.018.

### Relationship between the genotype distribution of rs430397 G>A and the status of HBeAg and immune response

Among 496 patients with persistent HBV infection, 193 carried HBeAg in the serum. The genotype distribution of rs430397 was not significantly different between patients with and without HBeAg. However, compared with the allele A in phase I group (12.92%) and phase III group (14.36%), phase IV group had a significantly higher frequency of allele A (22.77%; *P* = 0.006 and *P* = 0.015). Though the distribution of genotype AA between the groups was similar, the genotype GG was significantly lower in phase IV group (60.40%) than those in phase I group (77.53%, *P* = 0.005) and those in phase III group (73.27%, *P* = 0.027). In addition, compared with phase I group, phase IV group had a significantly higher frequency of heterozygote AG (33.66% versus 19.10%, *P* = 0.008) (Table 3).

**Table 3:** Genotype distribution of rs430397 G>A in the fifth intron of the *GRP78* gene in HBV-infected individuals with different HBeAg states.

Variables	Phase In = 89, (%)	Phase IIn = 104, (%)	Phase IIIn = 101, (%)	Phase IVn = 202, (%)
Age (mean ± SD, years)	34.8±9.5	42.4±12.6	37.9±10.3	46.5±14.8
Gender				
Female	38 (42.70)	41 (39.42)	44 (43.56)	70 (34.65)
Male	51 (57.30)	63 (60.58)	57 (56.44)	132 (65.35)
HBsAg state	+	+	+	+
HBeAg state	+	+	−	−
Clinical diagnosis	CHC	CHB or LC	IHC	CHB or LC
rs430397				
Alleles				
G	155 (87.08)	172 (82.69)	173 (85.64)	312 (77.23)
A	23 (12.92)	36 (17.31)	29 (14.36)	92 (22.77)*
Genotypes				
GG	69 (77.53)	72 (69.23)	74 (73.27)	122 (60.40)†
AG	17 (19.10)	28 (26.92)	25 (24.75)	68 (33.66)‡
AA	3 (3.37)	4 (3.85)	2 (1.98)	12 (5.94)

CHB, chronic hepatitis B; CHC, chronic hepatitis B virus carrier; HBeAg, hepatitis B e antigen; HBV, hepatitis B virus; IHC, inactive hepatitis B surface antigen carrier; LC, liver cirrhosis.

*Compared with the phase I group, *x^2^* = 7.562 and *P* = 0.006; and compared with the phase III group, *x^2^* = 5.969 and *P* = 0.015.

† Compared with the phase I group, *x^2^* = 8.039 and *P* = 0.005; and compared with the phase III group, *x^2^* = 4.883 and *P* = 0.027.

‡ Compared with the phase I group, *x^2^* = 7.131 and *P* = 0.008.

### Relationship between rs430397 genotype distribution and liver function profiles in patients with LC

All variables of interest, including liver function test results were listed in Table 4. The genotype distribution of rs430397 had no influence on common liver function, including total bilirubin (T-Bil), ALT, aspartate aminotransferase (AST) and γ-glutamyltransferase (GGT). But compared with the LC patients carrying GG genotype, those patients carrying AA genotype had a significantly lower ALB (23.6±6.9 g/L versus 27.1±6.7 g/L, *P* = 0.029) and a significantly higher PT (29.0±6.7 s versus 22.7±6.5 s, *P* = 0.016).

**Table 4:** Relationship between rs430397 G>A distribution and liver functional profiles in patients with liver cirrhosis.

Demographic data and main liver function profiles	GGn = 119	AGn = 66	AAn = 13
Age (mean ± SD, years)	42.2±11.7	43.5±10.2	45.1±12.0
Gender (female/male)	49/70	21/45	4/9
HBeAg-positive cases (n, %)	50 (42.02)	21 (31.82)	4 (30.77)
HBV-DNA level (lg copies/mL)	4.12±1.85	5.96±2.33	6.32±1.25
T-Bil (µmol/L)	192.4±169.1	151.6±124.9	170.6±128.9
ALB (g/L)	27.1±6.7	24.9±5.8	23.6±6.9*
ALT (IU/L)	170.8±145.9	145.3±119.3	158.8±135.3
AST (IU/L)	133.0±101.6	139.8±99.9	159.5±127.8
GGT (IU/L)	92.0±22.5	78.5±30.1	71.4±46.3
PT (seconds)	22.7±6.5	26.3±6.0	29.0±6.7†

SD, standard deviation; T-Bil, total bilirubin; ALB, albumin; ALT, alanine aminotransferase; AST, aspartate aminotransferase; GGT, γ-glutamyltransferase; PT, prothrombin time.

*Compared with the GG group, *x^2^* = 4.461 and *P* = 0.029.

† Compared with the GG group, *x^2^* = 5.832 and *P* = 0.016.

### Risk factors for the development of advanced cirrhosis in HBV patients

To assess the possible association between the polymorphism and cirrhosis in HBV infection, the cases were divided into two groups based on the absence or presence of cirrhosis. Significant association between the alleles (*P* = 0.001) and their genotypes (*P* = 0.011 or *P* = 0.014) and increased risk of HBV-related cirrhosis was found. The risk of cirrhosis increased with a dose-dependent manner as rs430397A allele increased (*P*-trend = 0.005; Table 5).

**Table 5:** rs430397 G>A polymorphism and cirrhosis risk in HBV patients.

Alleles/Genotypes	Noncirrhosisn = 298, (%)	Cirrhosisn = 198, (%)	OR (95%CI)*	*P* *
G	508 (85.23)	304 (76.77)	1	
A	88 (14.77)	92 (23.23)	1.75 (1.26–2.42)	0.001
GG	218 (73.15)	119 (60.10)	1	
AG	72 (24.16)	66 (33.33)	1.68 (1.12–2.51)	0.011
AA	8 (2.68)	13 (6.57)	2.98 (1.20–7.39)	0.014
*P*-trend†			0.005	

*Pearson Chi-square test.

† Multivariable logistic regression analysis adjusted with age and gender.

When the LC patients were divided using CPS, the distributions of age, gender and positive HBeAg did not differ significantly between the patients with advanced LC (CPS classes C and B) and those with mild LC (CPS class A; *P*>0.05 in each parameter), whilst the distributions of rs430397 alleles A and genotype AA were noted to be significantly different between these two groups (*P* = 0.010 or *P* = 0.032). The results of multivariable logistic regression analysis demonstrated that the rs430397 genotype did constitute a risk factor for the development of advanced LC (*P*-trend = 0.021; Table 6).

**Table 6:** Comparison between advanced and mild liver cirrhosis patients according to Child-Pugh score.

Parameters	A§	C+B§	*P*
Age (years, mean±SD)	41.8±10.6	44.5±11.9	0.559*
Gender			
Female	23 (53.49)	51 (32.90)	
Male	20 (46.51)	104 (67.10)	0.162†
HBeAg positive	20 (46.51)	55 (35.48)	0.187†
rs430397			
Alleles			
G	75 (87.21)	229 (73.87)	
A	11 (12.79)	81 (26.13)	0.010†
Genotypes			
GG	32 (74.42)	87 (56.13)	
AG	11 (25.25)	55 (35.48)	0.115†
AA	0 (0.00)	13 (8.39)	0.032†
*P*-trend			0.021‡

*Mann-Whitney U-test.

† Pearson Chi-square test.

‡ Multivariable logistic regression analysis adjusted with age and gender.

§ Child-Pugh classification.

## Discussion

Host genetic factors are widely viewed as the common basis of the different outcomes of HBV infection [28], [29], [30]. Our current study demonstrated a statistical relationship of rs430397 polymorphism of *GRP78* gene with HBV-related LC. Compared with individuals with non-HBV-infection or self-limited-infection, patients with LC were significantly more likely to carry allele A. For the genotype, the proportion of the AA genotype in patients with LC was higher than that in non-HBV-infected individuals. This finding indicated that patients carry allele A or genotype AA have an increased risk of LC compared with those carry allele G or genotype GG. In addition, logistic regression analysis showed that rs430397 genotype had an increased susceptibility to LC with a dose-dependent manner.

The functional severity of LC is usually described by CPS [31]. According to CPS, patients of LC in the present study were classified into calss A (mild), class B (moderate) and class C (severe). Notably, allele A and genotype AA constitute risk factors for the progression of advanced LC. It may suggest that rs430397 may also be a determinant for the degree of LC severity.

Although the genotype distribution of rs430397 was not significantly different between patients with and without HBeAg, when taking immune response phases of infection into account, the frequencies of allele A and heterozygote AG were significantly higher in phase IV group (HBeAg negative CHB or LC) compared with phase I group (chronic HBV carrier) and phase III group (inactive hepatitis B surface antigen carrier). This result does not reject the effect of rs430397 G>A on HBeAg maturation or seroconversion. Rather, HBV infected patients with allele A and heterozygote AG have a higher risk of developing HBeAg-negative CHB and LC. It was verified subsequently as we demonstrated a statistical relationship of the rs430397 at allele and heterozygote level with the progress of HBV infection.

Clinically, HBV infection does not invariably result in chronic hepatitis since the host possesses the ability to eliminate the virus spontaneously in most cases [32]. In acute infection, there is a persistence of HBsAg, HBeAg, and HBV-DNA [33]. After the acute hepatitis resolves, serum transaminases levels usually fall but may remain abnormal. There may be a dectectable anti-HBc IgM for the initial 6 months of infection that is later replaced by IgG. Over time, there may be seroconversion as defined by a loss of HBeAg and development of anti-HBe. Seroconversion is usually preceded by a marked decrease in serum HBV-DNA. Seroconversion may be associated with a flare in serum ALT levels but these levels typically decrease and normalize after seroconversion [34].

To decrease the bias of age and gender on the effect of estimates, we conducted multivariable logistic regression analysis and the association between rs430397 and the progression of HBV infection remained significant. Among multiple parameters evaluating liver function, ALB is quantitatively the most important of several plasma proteins formed in the liver [35]. Accordingly, measurement of total concentration of ALB is a useful test of hepatic synthetic function. The relatively long half-life of ALB makes the albumin level a better index of severity and prognosis in patients with chronic liver disease such as LC [36]. And also, these diseases are the common cause of impaired coagulation which was reflected by prolonged PT [37]. In patients with HBV-related LC, we found patients carrying AA genotype had relatively lower ALB levels and higher PTs compared with those carrying GG genotype. This fact further supports the potential association of rs430397 with the outcome of HBV infection.

Increasing evidence demonstrated that HBV infection is associated with oxidative stress, which also may cause structural damage to DNA or impede DNA repair process [38], [39]. The pre-S proteins are retained in the endoplasmic reticulum (ER) and subsequently induce ER stress, leading to the expression of ER chaperone GRP78. Accumulation of GRP78 induces ER stress signaling pathway, including unfolded protein response (UPR) and ER overload response [40]. In addition, cells infected by viruses are usually killed by a variety of reactive oxygen species (ROS), which are produced by Kupffer and inflammatory cells [41]. Unfortunately, through these defense mechanisms, oxidative stress may become inevitable and result in oxidative damage to DNA or other macromolecules. Chronic inflammation caused by HBV induced the release of free radicals, cytokines and chemokines resulting in cell proliferation, DNA damage, fibrosis and angiogenesis, and frequently associated with increased hepatocarcinogenesis risk [39]. Cirrhosis may also indicate pathological and genetic changes throughout the liver leading to a field cancerization effect, which increases the risk of hepatocarcinogenesis. GRP78 pathway is one of the most important responders to disease-associated stress [42]. Therefore, rs430397 polymorphism of *GRP78* gene in the present study might be owing to, at least partially, genetic instability induced by HBV-related cirrhosis.

Our study was limited by the small number of patients, but there would be some concern over the ethics of enrolling further large numbers of patients who were follow-up information on patient survival outcomes. Taken together, the present investigation first revealed a contributing factor (rs430397 within GRP78 gene) to LC in persistent HBV carriers in Chinese *Han* population.
